# Preoperatively staging liver fibrosis using noninvasive method in Hepatitis B virus-infected hepatocellular carcinoma patients

**DOI:** 10.18632/oncotarget.14024

**Published:** 2016-12-19

**Authors:** Hengyi Gao, Feng Zhu, Min Wang, Hang Zhang, Dawei Ye, Jiayin Yang, Li Jiang, Chang Liu, Renyi Qin, Lunan Yan, Guangqin Xiao

**Affiliations:** ^1^ Department of General Surgery, Guizhou Provincial People’s Hospital, Guiyang, China; ^2^ Department of Hepato-Pancreato-Biliary Surgery, Tongji Hospital of Tongji Medical College, Huazhong University of Science and Technology, Wuhan, China; ^3^ Department of Oncology, Tongji Hospital of Tongji Medical College, Huazhong University of Science and Technology, Wuhan, China; ^4^ Department of Liver Surgery, West China Hospital of Sichuan University, Chengdu, China; ^5^ Department of State Key Laboratory of Biotherapy and Pathology, West China Hospital of Sichuan University, Chengdu, China

**Keywords:** hepatocellular carcinoma, liver fibrosis, hepatitis B virus, preoperatively, noninvasive

## Abstract

**Background:**

Advanced liver fibrosis can result in serious complications (even patient’s death) after partial hepatectomy. Preoperatively percutaneous liver biopsy is an invasive and expensive method to assess liver fibrosis. We aim to establish a noninvasive model, on the basis of preoperative biomarkers, to predict liver fibrosis in hepatocellular carcinoma (HCC) patients with hepatitis B virus (HBV) infection.

**Methods:**

The HBV-infected liver cancer patients who had received hepatectomy were retrospectively and prospectively enrolled in this study. Univariate analysis was used to compare the variables of the patients with mild to moderate liver fibrosis and with severe liver fibrosis. The significant factors were selected into binary logistic regression analysis. Factors determined to be significant were used to establish a noninvasive model. Then the diagnostic accuracy of this novel model was examined based on sensitivity, specificity and area under the receiver-operating characteristic curve (AUC).

**Results:**

This study included 2,176 HBV-infected HCC patients who had undergone partial hepatectomy (1,682 retrospective subjects and 494 prospective subjects). Regression analysis indicated that total bilirubin and prothrombin time had positive correlation with liver fibrosis. It also demonstrated that blood platelet count and fibrinogen had negative correlation with liver fibrosis. The AUC values of the model based on these four factors for predicting significant fibrosis, advanced fibrosis and cirrhosis were 0.79-0.83, 0.83-0.85 and 0.85-0.88, respectively.

**Conclusion:**

The results showed that this novel preoperative model was an excellent noninvasive method for assessing liver fibrosis in HBV-infected HCC patients.

## INTRODUCTION

The hepatocellular carcinoma (HCC) patients are often accompanied with different degrees of liver fibrosis [1,2]. Severe hepatic fibrosis or cirrhosis can lead to serious complications after partial hepatectomy, and even may cause patients’ death from liver failure [3]. Meanwhile, assessing the severity of liver fibrosis can help surgeons to predict the operation risk and to select the optimal surgical approaches (hepatectomy or liver transplantation) for HCC patients who need operation [3]. Therefore, it is extremely necessary to evaluate the stages of liver fibrosis for HCC patients before surgery.

Currently, getting liver tissue by percutaneous liver biopsy or liver excision is still the traditional method for the assessment of liver fibrosis. However it is an invasive and expensive procedure which restricts its widespread implementation in clinical practice, especially in the areas of poor medical conditions. This invasive method makes patients having heavy pain feeling, bleeding, and even death. Some authors have suggested that pain occurs in 40% of patients, and the rate of death resulting from heavy bleeding has been relatively high during liver biopsy [4,5]. Although ultrasound-guided liver biopsy has reduced the incidence of heavy hemmorage and mortality, for the less experienced doctors these serious complications make both patients and physicians extremely anxious. Sampling error can also occur, particularly when the biopsy sample is small. A very important limitation of liver biopsy is that it is not an ideal method for dynamically monitoring liver fibrosis and cirrhosis. Thus it is absolutely essential to propose a reliable, simple and noninvasive method for assessing liver fibrosis.

Some researchers have attempted to establish models using biochemical variables of simple blood tests for the assessment of liver fibrosis. In the original studies performed by Wai [6] and Sterling [7], the researchers reported that the diagnostic values of the aspartate aminotransferase to platelet ratio index (APRI) and the fibrosis index based on four factors (FIB-4) were well-pleasing for distinguishing different stages of liver fibrosis. However, some authors have reported that these models are not very robust for evaluating liver fibrosis in HBV-positive patients [8]. Our previous study demonstrated that the accuracy of APRI and FIB-4 for predicting liver fibrosis related by HBV was moderate, which was different from the results of the original articles [9,10]. Another novel model was established based on 372 chronic HBV infection patients [11]. Because of needing special blood test variables and a small sample size, the application of this index has been limited. Based on our knowledge, no model which consists of simple blood tests factors has been published to preoperatively predict liver fibrosis in HBV-infected HCC patients. Given this we aim to propose a novel, simple and noninvasive model to preoperatively predict different levels of liver fibrosis for HCC patients with HBV infection.

## PATIENTS AND METHODS

### Patients selection

We obtained the data of liver cancer patients who underwent hepatectomy from the liver cancer database of West China Hospital (from January 2009 to March 2015). A portion of the data was collected retrospectively, and the others were collected prospectively. Briefly, eligibility criteria included age greater than 18 years old, infection with HBV, having liver cancer and receiving partial liver resection. The patients who lacked Ishak scores for liver fibrosis evaluation were excluded. The patients who were not HCC, having haematological disease or acute liver dysfunction were also excluded from this study.

The informed consent was obtained from each patient included in the study. The study protocol conformed to the ethical guidelines of the 1975 Declaration of Helsinki of the World Medical Association. The Institutional Review Board of the West China Hospital of Sichuan University in Sichuan Province approved this study. The patients were represented in the study by code numbers, and their personal data were concealed.

### Blood tests and fibrosis assessment

The blood test results were recorded within three days before the patients underwent surgery. Commonly used variables, including peripheral blood cell count, liver function, kidney function and blood coagulation function, were employed. The parameters of the peripheral blood cell count test included the white blood cell count (WBC), platelet count (PLT) and hemoglobin level. The indices reflecting liver and kidney function included aspartate aminotransferase (AST), alanine aminotransferase (ALT), alkaline phosphatase (ALP), γ-glutamyl endopeptidase (GGT), total bilirubin (TBL), direct bilirubin (DBL), total protein (TP), albumin (Alb), globulin (Glo), blood urea nitrogen (BUN), serum creatinine (Scr) and cystatin C. The blood coagulation indices included prothrombin time (PT), activated partial thromboplastin time (APTT), international normalized ratio (INR) and fibrinogen (FIB).

The pathological evaluation of the excised liver tissue was considered as the gold standard for tumor and non-neoplastic liver tissue assessment. The tumor(s) and the liver tissue away from tumors were resected and immediately sent for tumor diagnosis and fibrosis assessment by two pathologists who received the same training. The discrepancies between the two pathologists were resolved by their collaborative discussion and consensus. The pathologists were blinded to the patients’ characteristics. The interval time between liver fibrosis assessment and blood tests was less than 1 week. In order to ensure the sample was sufficiently large, the size of the non-neoplastic liver tissue for fibrosis assessment was at least 1cm*1cm. Liver fibrosis stages were determined by the Ishak score system. Significant fibrosis, advanced fibrosis and cirrhosis were defined as Ishak score of 3-6, 4-6 and 5-6, respectively.

### Statistical analysis

The statistical analysis was performed using SPSS version 17.0 (SPSS Inc, Chicago, Illinois, USA). The independent sample *t*-test and Pearson's chi-square (χ2) test were used to analyze the differences in variables between patients in retrospective and prospective cohorts. We compared the demographic and clinical data of the groups with different levels of liver fibrosis. In addition, the variables in the retrospective cohort that showed significant differences were selected for binary regression analysis. According to the results of the regression analysis, a new model for assessing liver fibrosis was established. Then, three optimal cut-off values were determined by receiver-operating characteristic curve (ROC) analysis. Four-fold tables (2×2) were constructed to calculate the sensitivity, specificity, positive predictive value (PPV), negative predictive value (NPV) and diagnostic coincidence rate (DCR) of the novel index for assessing liver fibrosis and cirrhosis. The area under the ROC curve (AUC) analysis of this model for detecting stages of liver fibrosis was validated in prospective cohort and total cohort. The data was presented as the mean values ± standard deviation (SD). The statistically significant differences were defined as P<0.05.

## RESULTS

### Patient characteristics

The 2,291 subjects from the liver cancer database of West China Hospital were enrolled in the study (including 1,765 retrospective subjects and 526 prospective subjects). Eight patients were younger than 18 years old. Sixty five patients were excluded for non-HCC assessed by pathology. Twenty seven patients lacked the results of liver fibrosis assessment in pathological reports. Six patients had haematological diseases and 9 patients had acute liver dysfunction. A total of 115 patients were excluded from this study. Finally, this study included 2,176 HBV-infected HCC patients who had undergone hepatectomy (1,682 retrospective subjects and 494 prospective subjects). The demographic and clinical characteristics of all included patients were shown in Table 1. In the total cohort the male patients accounted for 85.5%. The mean age of all patients was 50.8 (SD:11.6) years old. Table 1 showed the proportion of patients with different liver fibrosis stages. It demonstrated that there was statistical difference only in the distribution of the different levels of liver fibrosis between retrospective and prospective cohorts.

**Table 1:** Patient characteristics at baseline

Variables	All Patients	Retrospective Cohort	Prospective Cohort	*P* value
Number of patients	2176	1682	494	-
Male (%)	1860 (85.5%)	1445 (85.9%)	415 (84.0%)	0.29
Age (years)	50.8±11.6	50.7±11.8	51.4±11.3	0.70
Weight (kg)	62.4±9.8	62.3±9.7	63.0±10.3	0.21
BMI	22.6±2.6	22.5±2.5	22.8±2.8	0.19
Hemoglobin (g/L)	140.5±2.4	139.3±20.9	144.6±18.1	0.34
Platelet (10^9^/L)	140±72	136±71	143±73	0.52
WBC (10^9^/L)	5.9±2.8	5.9±2.9	5.9±2.5	0.52
TBL (μmol/L)	20.4±37.6	20.5±36.5	20.0±37.3	0.84
DBL (μmol/L)	9.3±25.7	9.5±27.7	8.8±24.9	0.23
AST (IU/L)	56.5±70.9	57.5±78.3	54.4±72.2	0.27
ALT (IU/L)	57.6±98.3	59.7±99.2	56.4±97.3	0.33
AST/ALT	1.2±0.9	1.2±0.7	1.2±0.9	0.19
ALP (IU/L)	114.2±88.6	113.7±89.9	116.0±84.2	0.61
GGT (IU/L)	110.0±149.3	108.1±137.3	116.3±143.2	0.36
Albumin (g/L)	40.3±5.5	40.1±5.8	40.9±4.3	0.78
Globulin (g/L)	29.0±5.5	28.8±5.5	29.3±5.3	0.56
Total protein (g/L)	69.2±7.3	68.9±7.7	70.3±6.1	0.34
BUN (mmol/L)	5.9±3.8	6.0±3.4	5.7±3.7	0.70
Scr (μmol/L)	76.2±20.6	76.6±18.9	74.9±25.5	0.18
Cystatin C (μg/dl)	105.2±20.9	106.0±21.8	104.5±19.8	0.27
PT (s)	12.4±2.2	12.4±2.5	12.3±2.2	0.82
APTT (s)	31.5±6.8	31.8±9.1	30.4±4.6	0.74
INR	1.12±0.33	1.12±0.38	1.11±0.10	0.32
FIB (mg/cl)	29.9±13.6	29.8±14.1	28.9±11.0	0.21
Ishak score: 0-1 (%)	28 (1.3%)	21 (1.2%)	7 (1.4%)	<0.001*
Ishak score: 2 (%)	82 (3.8%)	49 (2.9%)	33 (6.7%)	
Ishak score: 3 (%)	262 (12.0%)	194 (11.5%)	68 (13.8%)	
Ishak score: 4 (%)	327 (15.0%)	235 (14.0%)	92 (18.6%)	
Ishak score: 5 (%)	365 (16.8%)	246 (14.6%)	119 (24.1%)	
Ishak score: 6 (%)	1112 (51.1%)	937 (55.7%)	175 (35.4%)	

* significant *P* value.

### Univariate and multivariate analysis

We conducted the univariate analysis to compare the parameters of the patients with mild to moderate fibrosis (F0-F4) and cirrhosis (F5-F6) in the retrospective cohort and prospective cohort, respectively. The results were shown in Table 2. It indicated that 9 biochemical parameters (platelet, WBC, TBL, DBL, AST, ALT, PT, INR and FIB) were significantly different between patients with lower Ishak scores and higher Ishak scores in retrospective cohort and prospective cohort. Table 2 demonstrated that the platelet count (P<0.001), WBC count (P<0.001) and serum FIB level (P<0.001) of the patients with severe liver fibrosis were markedly lower than those of the patients with mild to moderate liver fibrosis. The biochemical markers reflecting liver function (including TBL, DBL, AST and ALT) were obviously elevated in the patients with higher Ishak scores. The results showed that two coagulation function indexes (PT and INR) of the patients with cirrhosis increased visibly, with all P values less than 0.001.

**Table 2:** Comparison of the demographic and clinical characteristics of patients at different fibrosis stages in the retrospective and prospective cohorts

Variables	Retrospective Cohort			Prospective Cohort			
	Ishak score: 0-4	Ishak score: 5-6	*P* value		Ishak score: 0-4	Ishak score: 5-6	*P* value
Number of patients	499	1183	-		200	294	-
Male (%)	412(82.5%)	1033(87.3%)	0.01*		163(81.5%)	252(85.7%)	0.21
Age (years)	51.9±12.4	50.2±11.4	0.06		52.4±12.7	50.7±10.2	0.19
Weight (kg)	61.2±9.5	62.8±9.7	0.11		62.8±10.6	63.0±10.1	0.77
BMI	22.4±2.4	22.7±2.6	0.17		22.9±3.1	22.7±2.6	0.56
Hemoglobin (g/L)	140.3±19.1	139.0±21.6	0.22		142.9±18.0	145.8±18.1	0.08
Platelet (10^9/L)	170±75	123±64	<0.001*		182±73	130±64	<0.001*
WBC (10^9/L)	6.3±2.3	5.7±3.1	<0.001*		6.3±2.3	5.7±2.6	0.02*
TBL (μmol/L)	15.3±10.7	22.7±42.9	<0.001*		14.7±11.1	23.5±31.4	<0.001*
DBL (μmol/L)	6.0±7.5	10.9±32.5	<0.001*		6.0±5.6	7.3±19.3	0.02*
AST (IU/L)	54.8±60.7	64.4±91.4	0.03*		54.7±57.9	62.9±74.2	0.04*
ALT (IU/L)	54.3±66.5	62.0±72.0	0.02*		50.2±59.4	60.5±68.2	0.01*
AST/ALT	1.20±0.68	1.18±0.72	0.60		1.3±1.2	1.2±1.8	0.54
ALP (IU/L)	114.4±103.6	113.4±83.4	0.84		126.5±101.3	118.7±97.4	0.23
GGT (IU/L)	112.2±158.6	106.4±127.3	0.43		133.4±128.6	124.7±118.8	0.09
Albumin (g/L)	40.5±5.5	40.0±6.0	0.34		40.9±4.6	40.9±4.2	0.98
Globulin (g/L)	28.1±5.0	29.1±5.8	0.15		29.4±5.3	29.4±5.4	0.99
Total protein (g/L)	68.6±7.4	69.0±7.8	0.42		70.3±6.6	70.3±5.8	0.94
BUN (mmol/L)	5.4±1.7	6.2±1.9	0.37		5.8±2.9	5.6±4.1	0.60
Scr (μmol/L)	75.1±15.5	77.2±20.2	0.39		76.0±27.2	74.2±24.2	0.45
Cystatin C (μg/dl)	110.6±25.8	112.7±28.4	0.44		108.4±23.9	117.8±26.8	0.74
PT (s)	11.8±1.4	12.6±2.8	<0.001*		11.3±1.1	12.5±1.1	<0.001*
APTT (s)	28.1±6.5	33.4±10.9	0.28		29.7±4.7	30.8±4.5	0.18
INR	1.06±0.13	1.15±0.44	<0.001*		1.08±0.09	1.12±0.10	<0.001*
FIB (mg/cl)	33.9±17.1	28.7±12.7	<0.001*		32.3±11.8	26.5±9.7	<0.001*

* significant *P* value.

Then, the 9 variables of the retrospective cohort were selected into binary regression analysis. Table 3 displayed the results of the multivariate analysis. Based on the results, the following four variables were selected for the equation: PLT, TBL, PT and FIB. According to the degrees of the association between the four variables and the different stages of liver fibrosis, we proposed a model to predict liver fibrosis using these four factors. And we refer to this model as the LivFib index. The model was calculated as follows:

**Table 3:** Binary logistic analysis identifying independent factors associated with fibrosis staging (Ishak 0-4 vs. 5-6) in retrospective cohort

Variables	Wald	Odds Ratio	95% CI^#^	*P* value
Platelet (10^9/L)	74.8	0.992	0.990-0.994	<0.001*
TBL (μmol/L)	7.49	1.013	1.004-1.023	0.006*
PT (s)	32.8	1.340	1.212-1.481	<0.001*
FIB (mg/cl)	6.4	0.987	0.978-0.997	0.011*

# CI, confidence interval; * significant *P* value.

LivFib index: (TBL×PT×100)/(PLT×FIB)

* TBL, mol/L; PT, s; PLT, 10^9/L; FIB, mg/cl.

### Examination of the model

**Figure 1:**
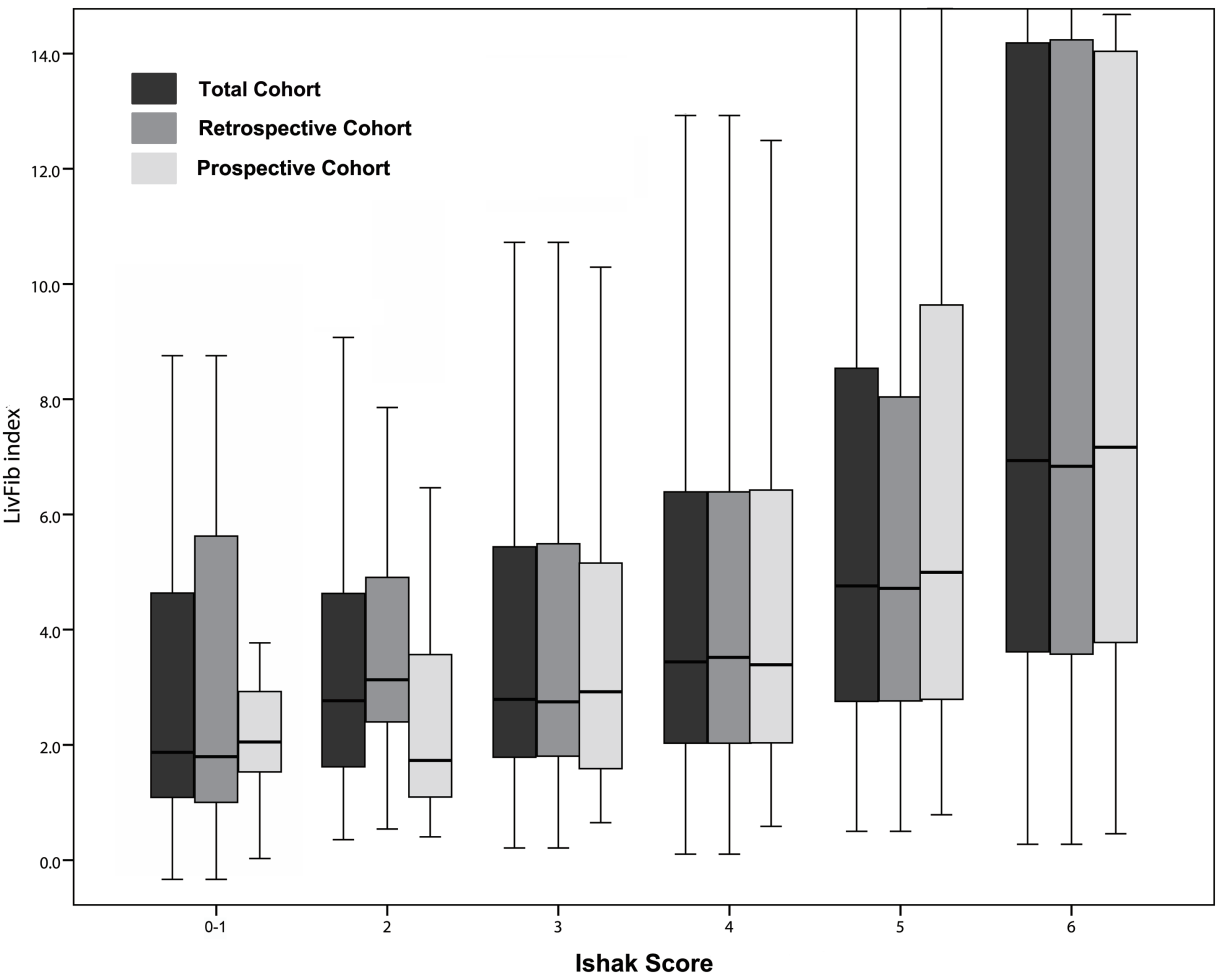
Box plot of LivFib Index scores distributing at different fibrosis stages

**Figure 2:**
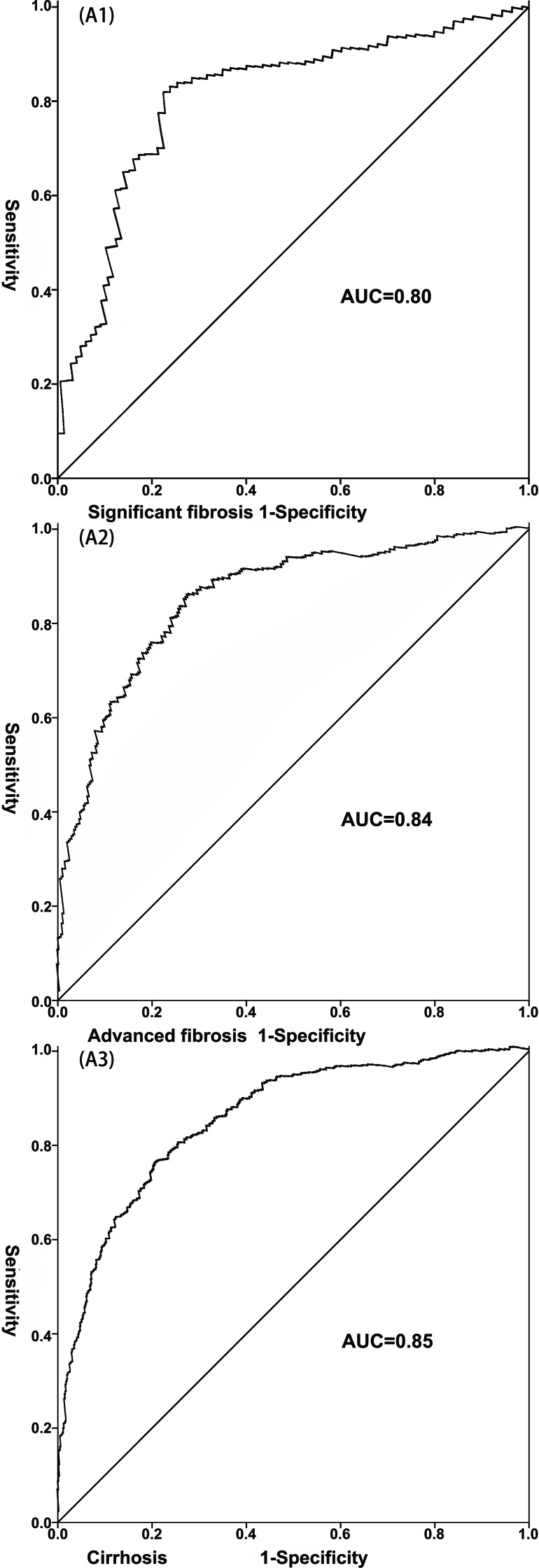
The receiver-operating characteristic curve (ROC) of the LivFib Index for predicting liver fibrosis in retrospective cohort (**A1**) The ROC of the LivFib Index for predicting significant fibrosis; (**A2**) The ROC of the LivFib Index for predicting advanced fibrosis; (**A3**) The ROC of the LivFib Index for predicting cirrhosis.

After the model was established, each of the subjects received a corresponding LivFib index value. Figure 1 showed the distribution of the index values in the total cohort, retrospective cohort and prospective cohort at different stages of liver fibrosis. We found that LivFib index values present the ascending trend with the increase of the Ishak scores. The mean LivFib index values of the patients with fibrosis stages of F5-F6 were 19.0 ± 4.7, 19.2 ± 4.8 and 18.9 ± 4.4 in the total cohort, retrospective cohort and prospective cohort, respectively. We found that the LivFib index values of the patients with cirrhosis were visibly higher than those of the patients with mild and moderate fibrosis (with all P values less than 0.001). Figure 2 showed the ROC curve analysis of the retrospective cohort. We obtained three ideal cut-off values (3.0, 4.0 and 5.0) for this model to predict different levels of fibrosis. The AUC values of LivFib index were 0.80, 0.84 and 0.85 for diagnosing significant fibrosis, advanced fibrosis and cirrhosis in retrospective cohort.

**Figure 3:**
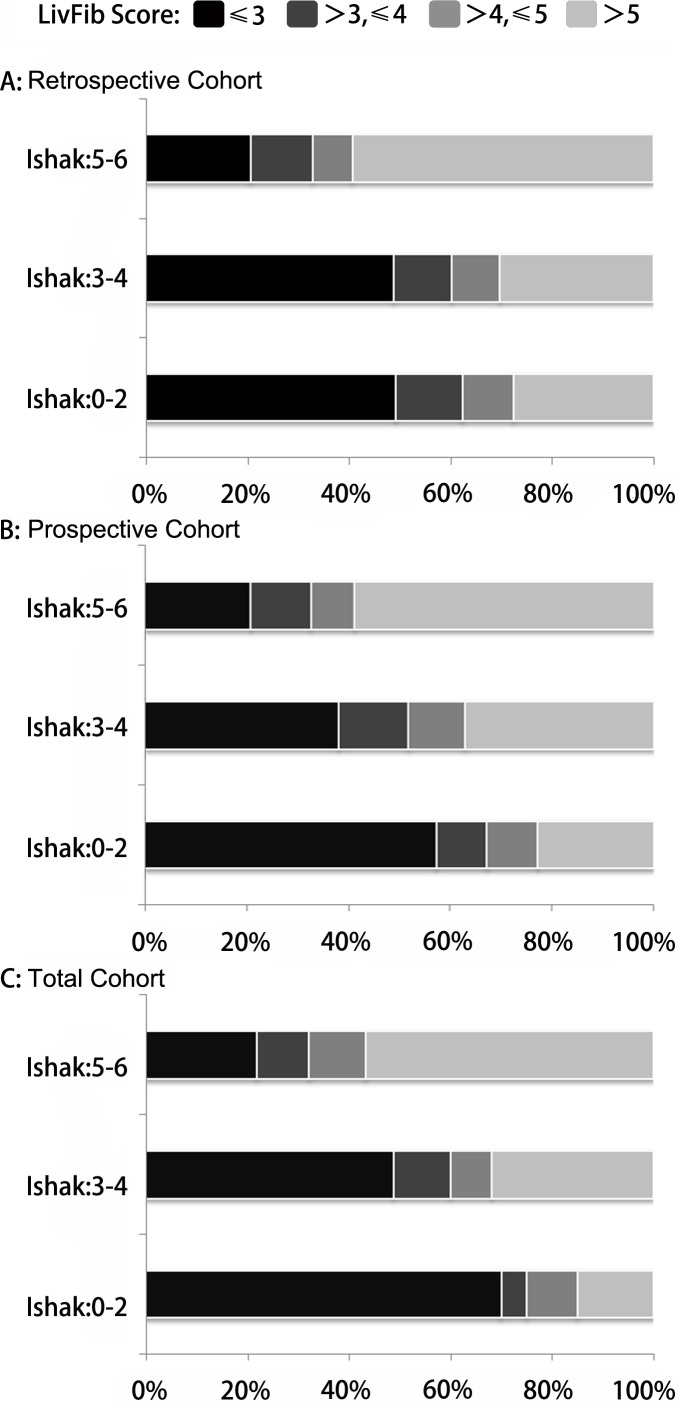
The percentages of patients with different LivFib Index scores (≤3.0, 3.0-4.0, 4.0-5.0 and >5.0) at different fibrosis stages **A**. The percentages of patients with different LivFib Index scores at significant fibrosis, advanced fibrosis and cirrhosis in retrospective cohort; **B**. The percentages of patients with different LivFib Index scores at significant fibrosis, advanced fibrosis and cirrhosis in prospective cohort; **C**. The percentages of patients with different LivFib Index scores at significant fibrosis, advanced fibrosis and cirrhosis in the total cohort.

Then the subjects of the retrospective cohort, prospective cohort and total cohort were respectively divided into four groups by the cut-off values. Figure 3 displayed the distribution of the patients with different index values in significant fibrosis category, advanced fibrosis category and cirrhosis category. It indicated that the proportion of the patients with index value less than 3 was smallest in the cirrhosis category and the patients with index value more than 5 occupied the greatest proportion in the cirrhosis category. In the advanced fibrosis category, the proportion of the patients with index value more than 5 was greater than that in the significant fibrosis category and was smaller than that in the cirrhosis category. In the advanced fibrosis group, the proportion of the patients with index value less than 3 was smaller than that in the significant fibrosis group and was greater than that in the cirrhosis group.

**Figure 4:**
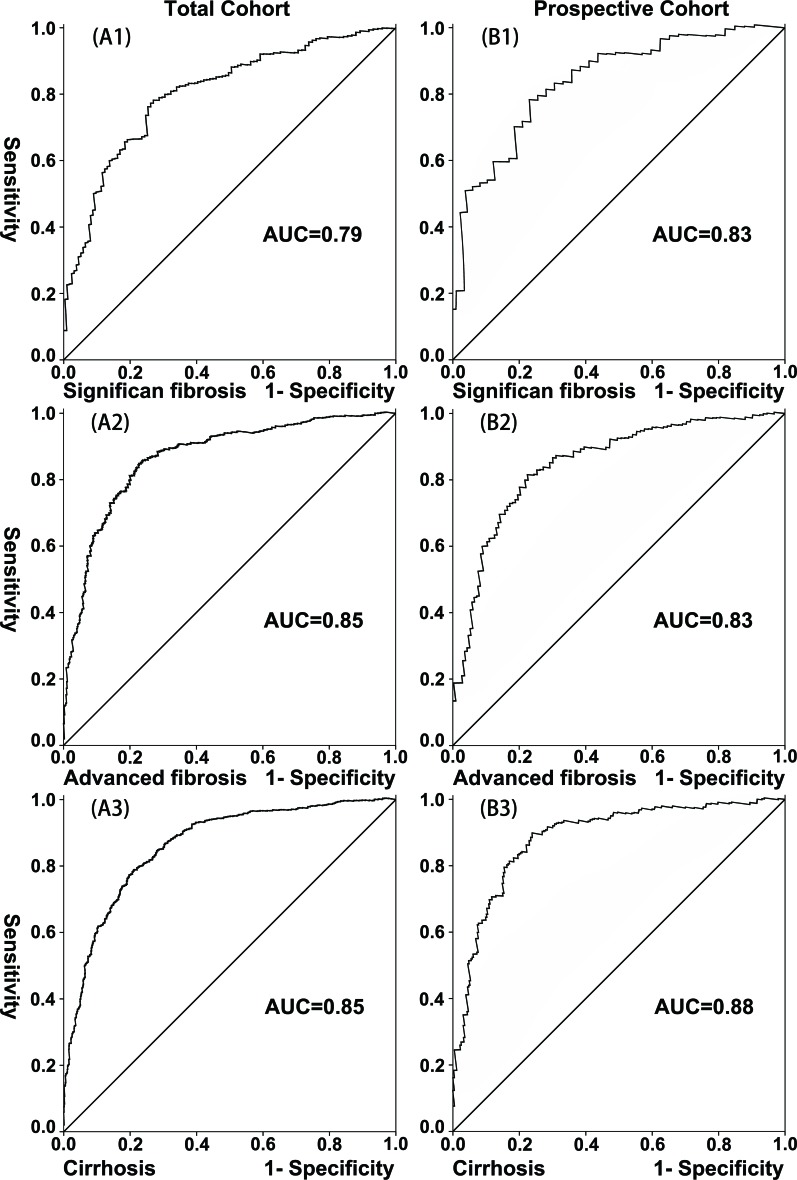
Validation of the LivFib Index for predicting liver fibrosis by the receiver-operating characteristic curve (ROC) in the total cohort and prospective cohort (**A1**, **A2** and **A3**) The ROC of the LivFib Index for predicting significant fibrosis, advanced fibrosis and cirrhosis in the total cohort; (**B1**, **B2** and **B3**) The ROC of the LivFib Index for predicting significant fibrosis, advanced fibrosis and cirrhosis in prospective cohort.

Next, we examined the diagnostic utility of this model by ROC analysis in the total cohort and prospective cohort, as showed in Figure 4. The AUC values of the LivFib index ranged from 0.79 to 0.83 for detecting significant fibrosis. The AUC values ranged from 0.83 to 0.85 for diagnosing advanced fibrosis and from 0.85 to 0.88 for predicting cirrhosis. Table 4 displayed the diagnostic accuracy of the LivFib Index for predicting varying degrees of liver fibrosis. At the cut-off of 3.0, the sensitivities ranged from 90.4% to 93.0%, 93.6% to 94.6% and 95.6% to 96.1% for predicting significant fibrosis, advanced fibrosis and cirrhosis. At the threshold of 5.0, the specificities were 77.9% to 94.3%, 92.5% to 93.4% and 90.9% to 91.2% for diagnosing significant fibrosis, advanced fibrosis and cirrhosis. The highest PPV value was 99.1% and lowest NPV value was 3.9%. Meanwhile, Table 4 exhibited the DCR values of this model for detecting liver fibrosis, and the highest DCR value was 91.6%.

**Table 4:** The diagnostic accuracy of LivFib index for predicting significant fibrosis, advanced fibrosis and cirrhosis in retrospective cohort, prospective cohort and total cohort

	Cut-off values	Positive (*n*)	Negative (*n*)	Sensitivity	Specificity	PPV#	NPV#	DCR#
**Significant Fibrosis (Ishak: 0-2 vs. 3-6)**								
Retrospective Cohort	>3.0	1902	71	91.2%	21.4%	96.4%	9.6%	88.3%
	>4.0	1227	27	58.9%	70.0%	97.8%	6.9%	59.3%
	>5.0	538	5	25.8%	94.3%	99.0%	5.2%	28.7%
Prospective Cohort	>3.0	1447	68	90.4%	16.7%	95.5%	8.1%	86.8%
	>4.0	920	27	57.5%	66.7%	97.1%	7.4%	57.9%
	>5.0	405	6	25.3%	92.6%	98.5%	5.9%	28.6%
Total Cohort	>3.0	446	8	93.0%	43.8%	98.3%	15.6%	91.6%
	>4.0	296	3	61.6%	81.3%	99.1%	5.9%	62.1%
	>5.0	125	4	26.3%	77.9%	96.9%	3.9%	28.1%
**Advanced Fibrosis (Ishak: 0-3 vs. 4-6)**								
Retrospective Cohort	>3.0	1717	258	93.6%	24.3%	86.9%	41.3%	82.7%
	>4.0	1154	101	62.9%	70.3%	91.9%	26.1%	64.0%
	>5.0	519	25	28.3%	92.8%	95.5%	19.4%	38.4%
Prospective Cohort	>3.0	1242	322	94.3%	11.6%	79.4%	35.9%	76.4%
	>4.0	864	83	61.7%	70.6%	91.3%	27.0%	63.2%
	>5.0	390	21	27.8%	92.5%	94.9%	20.5%	38.7%
Total Cohort	>3.0	404	51	94.6%	23.7%	88.8%	40.9%	85.0%
	>4.0	278	20	65.1%	69.7%	93.2%	23.9%	65.7%
	>5.0	124	4	29.1%	93.4%	96.6%	17.1%	37.9%
**Cirrhosis (Ishak: 0-4 vs. 5-6)**								
Retrospective Cohort	>3.0	1462	513	95.6%	20.7%	74.0%	66.5%	73.3%
	>4.0	1039	215	67.9%	66.7%	82.8%	46.8%	67.6%
	>5.0	485	58	31.7%	91.0%	89.3%	36.0%	49.3%
Prospective Cohort	>3.0	1099	416	95.3%	21.4%	72.6%	67.6%	72.1%
	>4.0	769	178	66.7%	66.4%	81.2%	47.7%	66.6%
	>5.0	363	48	31.5%	90.9%	88.3%	37.8%	50.1%
Total Cohort	>3.0	350	105	96.1%	19.0%	76.9%	63.6%	75.9%
	>4.0	256	42	70.2%	67.3%	85.8%	44.6%	69.5%
	>5.0	117	11	32.2%	91.2%	91.1%	32.4%	47.7%

# PPV, Positive Predictive Value; NPV, Negative Predictive Value; DCR, Diagnostic Coincidence Rate.

## DISCUSSION

The objective of this study is to propose a novel noninvasive model based on preoperative biochemical markers to predict the stages of liver fibrosis in hepatocellular carcinoma patients with hepatitis B virus (HBV) infection. Although several researchers have establsihed models for predicting liver fibrosis caused by hepatitis C virus (HCV) or HCV/human immunodeficiency virus (HIV) co-infection [6,7,12,13]. However several researches have indicated that these models are not suitable for all patients with liver fibrosis induced by different etiologies [14,15]. For the patients with chronic HBV infection, early diagnosis of liver fibrosis is necessary because it can guide anti-HBV treatment, so as to delay the progress of disease. Meanwhile it is particularly important to assess the status of liver fibrosis for the HBV-infected HCC patients before surgery. Because preoperatively knowing the severity of liver fibrosis of these people can help surgeons to predict the risk of operation and the possible incidence of postoperative complications. Furthermore, knowing the liver fibrosis severity of the HCC patients plays a decisive role in the selection of the optimal radical treatments (including partial liver resection, liver transplantation or radiofrequency ablation).

So far, many methods have been proposed for noninvasive detection of liver fibrosis, such as liver transient elastography (Fibroscan) [16,17]. Research showed that the Fibroscan technique had a good value in the diagnosis of chronic HBV or HCV-related liver fibrosis [18,19]. Based on our knowledge, there is no preoperative noninvasive method on the basis of blood tests for the detection of liver fibrosis in HCC patients with HBV infection. Taken these issues into account, we summarized the data of 2,176 HBV-infected patients with HCC who underwent hepatectomy at West China Hospital. Our results demonstrated that four factors were associated with the degrees of liver fibrosis. A model consisting of these four variables was established after we performed a binary regression analysis. And the scores derived from the model were used to diagnose various levels of liver fibrosis. The analysis indicated that this model had robust utility in the diagnosis of liver fibrosis (Table 4, Figure 2 and Figure 4).

According to the univariate and multivariate analyses, we found that TBL and PT were positively correlated with the degrees of liver fibrosis; in contrast, PLT and FIB had negative relationships with the liver fibrosis stages. Some authors have suggested that the incidence of liver fibrosis in older people is higher or that they have more severe fibrosis compared to younger patients [7,20,21]. However, the results of our study demonstrated that the necessary links did not exist between age and liver fibrosis. Some researchers suggested that the increased serum AST and ALT were correlated with liver fibrosis and cirrhosis [6,7,11,13]. The authors hold the opinion that AST and ALT mainly exist in the plasma and mitochondria of liver cells [22,23]. The cellular and mitochondrial injury cause marked release of AST and ALT [24]. However our results indicated that serum AST and ALT levels had no association with liver fibrosis severity. Many studies have revealed that the elevation of certain indicators of hepatic function, such as serum bilirubin, suggests the presence of liver fibrosis [25,26]. Our results confirmed that there was correlation between TBL and liver fibrosis. But our results showed that ALT and AST were not associated with liver fibrosis stages. We also found that some indicators of coagulation were associated with the degrees of liver fibrosis, which is similar to the results of other researchers [27,28]. The PT was also selected into the equation, and one different thing was that our model included the serum FIB level.

The exact explanation for the elevated variables (TBL and PT) and the decreased plasma factors (PLT and FIB) in HBV-infected HCC patients who had high Ishak scores could not be clearly determined. We presented some possible hypothesis. Due to liver cell damage and the destruction of the liver lobule structure, bilirubin cannot be properly discharged into tiny bile ducts and subsequently reflux into blood, resulting in an increase of TBL[29]. The platelet count has been recognized to be correlated with the degree of portal hypertension induced by cirrhosis. The reduced platelet count in the peripheral blood and decreased thrombopoietin synthesis have also been considered to be relevant to hepatic function [30,31]. Similarly, PT values increase with the progression of liver fibrosis and the death of liver cells. At the same time, the original reduction in serum FIB, a protein synthesized in the liver, directly reflects liver cell injury and the decline of hepatic synthetic function.

We acknowledged that this study had several limitations. First, a large proportion of the patients had high Ishak scores. And the percentages of patients with different fibrosis levels in the total cohort, retrospective cohort and prospective cohort were different, which might had diminished the diagnostic accuracy of this model to a certain extent. In addition, the patients whose fibrosis assessment scores could not be found in the pathologic reports were excluded from this study. All the patients enrolled in this study had HCC, which might have affected the results. Additionally, the confounding factors including previous alcohol consumption and parasitic infection were not accurately assessed in this study. Moreover, we could not precisely determine the interval between the time when the patients became infected with HBV and the time when they underwent surgery. We caunnot obtain the accurate years that the patients had been infected with HBV. At last, the data of the anti-virus treatment for all HBV-infected patients could not be accurately recorded.

In conclusion, our results suggest that the LivFib index is a robust model for assessing liver fibrosis in HBV-infected HCC patients preoperatively. This model can be applied in the early diagnosis of liver fibrosis and cirrhosis. Future prospective and multicenter studies are needed to validate the utility of this model. In addition, further basic researches will be essential to reveal the mechanisms underlying the associations between the blood parameters and liver fibrosis stages.

### Abbreviations

HBV, hepatitis B virus; HCC, hepatocellular carcinoma; TBL, total bilirubin; APRI, aspartate aminotransferase to platelet ratio index; FIB-4, the fibrosis index based on four factors; WBC, white blood cell count; PLT, platelet count; AST, aspartate aminotransferase; ALT, alanine aminotransferase; ALP, alkaline phosphatase; GGT, γ-glutamyl endopeptidase; DBL, direct bilirubin; TP, total protein; Alb, albumin; Glo, globulin; BUN, blood urea nitrogen; Scr, serum creatinine; PT, prothrombin time; APTT, activated partial thromboplastin time; INR, international normalized ratio; FIB, fibrinogen; ROC, receiver-operating characteristic curve; PPV, positive predictive value; NPV, negative predictive value; DCR, diagnostic coincidence rate; AUC, area under the ROC curve; SD, standard deviation; HCV, hepatitis C virus; HIV, human immunodeficiency virus.
